# Features of Alteration in MAPK Pathway Activity in the Postnatal Brain of a Rat Model of Sporadic Alzheimer’s Disease

**DOI:** 10.3390/ijms27125430

**Published:** 2026-06-16

**Authors:** Natalia A. Muraleva, Natalia A. Stefanova, Nataliya G. Kolosova

**Affiliations:** Institute of Cytology and Genetics, Siberian Branch of Russian Academy of Sciences (ICG SB RAS), 10 Lavrentyeva Ave., Novosibirsk 630090, Russia; stefanovan@mail.ru (N.A.S.); kolosova@bionet.nsc.ru (N.G.K.)

**Keywords:** MAPK signaling, ERK1/2, JNK, p38 MAPK, brain development, hippocampus, prefrontal cortex, OXYS rats, neurodegeneration, Alzheimer’s disease

## Abstract

Early-life factors influence adult-brain vulnerability to sporadic Alzheimer’s disease (AD), but the underlying molecular mechanisms are unknown. In this study, we performed an integrated analysis of mitogen-activated protein kinases (MAPK) pathways’ (ERK1/2, JNK, and p38 MAPK) activity in the hippocampus and prefrontal cortex of OXYS rats (a model of sporadic AD) on postnatal days 3 and 10 (P3 and P10): critical periods of brain maturation. Wistar rats (healthy controls) showed extensive developmental transcriptional remodeling of all MAPK pathways. OXYS rats exhibited alterations, most pronounced in the prefrontal cortex at P3, with the JNK pathway showing the greatest divergence. At the protein level, OXYS rats failed to show the normal age-related increase in hippocampal ERK1/2 phosphorylation and in JNK1/2 levels in both regions, indicating developmental signaling deficits. p38 MAPK remained stable among Wistar and OXYS rats. Thus, delayed brain maturation, which contributes to accelerated brain aging and neurodegeneration in OXYS rats, occurs simultaneously with alterations in MAPK signaling. These aberrations potentially are able to increase brain susceptibility to age-related pathologies later in life.

## 1. Introduction

Age is the main risk factor for the sporadic form of AD: the most common progressive senile dementia. There are no effective methods to prevent and treat this neurodegenerative disease, owing to an incomplete understanding of its pathogenesis. There is growing evidence that environmental and genetic factors operating during critical developmental periods lead to increased susceptibility of the adult brain to neurodegenerative pathology in old age [1,2,3,4,5].

Epidemiological studies have linked perinatal factors, including hypoxia, nutritional status, maternal inflammation, and early-life stress, to an elevated risk of AD decades later [4,5,6]. Adverse early-life experiences can induce persistent alterations in synaptic plasticity, neurotrophic signaling, and stress responsiveness with aging [7,8]. Nonetheless, molecular mechanisms through which early-life events influence the neurodegeneration risk remain unknown. MAPKs represent evolutionarily conserved signaling cascades that regulate cellular responses to extracellular stimuli [9,10,11,12,13]. In mammals, three principal MAPK families have been characterized: extracellular signal-regulated kinases 1 and 2 (ERK1/2), p38 MAPKs, and c-Jun N-terminal kinases (JNK1/2/3) [6]. Each of them is a component of a three-tiered phosphorylation cascade (MAPKKK → MAPKK → MAPK) that regulates cellular responses through pathway-specific substrates [9,10,11,12,13].

Within the nervous system, MAPK signaling is important throughout the lifespan. During embryonic and early postnatal development, ERK1/2 orchestrate neurogenesis, neuronal migration, and differentiation downstream of neurotrophins and growth factors [14,15]. The stress-responsive p38 and JNK pathways, while traditionally associated with cellular stress responses, also contribute to normal neurodevelopment by modulating cytoskeletal dynamics, axon guidance, and synaptic formation [16,17,18,19,20]. In the mature brain, these cascades subserve synaptic plasticity, long-term potentiation, memory consolidation, and neuronal survival [16,21,22,23,24]. Nevertheless, when dysregulated—particularly under conditions of chronic oxidative stress—the same pathways can promote neurodegeneration, with excessive JNK and p38 activation contributing to tau hyperphosphorylation, amyloid-β toxicity, and apoptotic cell death [25,26,27,28,29]. A growing body of evidence indicates that the development of AD and other neurodegenerative diseases is associated with the activation of MAPK pathways; however, there is no information on changes in the activity of MAPK signaling pathways during brain maturation and whether these changes can affect the development of AD later in life. Here we assessed alterations in MAPK signaling in the developing brain of senescence-accelerated OXYS rats. They are a suitable model of sporadic AD and spontaneously develop key signs of AD: a progressive cognitive decline, amyloid-β accumulation, a tau pathology, and mitochondrial and synaptic dysfunction without additional impacts [30]. We have previously demonstrated that manifestation and progression of the AD-like pathology in OXYS rats occur concurrently with enhanced activation of all three MAPK cascades [31,32,33]. On postnatal day 20 (P20), however, when rat brain maturation is complete, no differences in MAPK signaling activity were detected (in our studies) in the cerebral cortex and hippocampus of OXYS rats and control (Wistar) rats.

An important role in the accelerated aging of the OXYS rats’ brain and in the development of AD signs in these animals is played by the delayed brain maturation associated with insufficient glial support; this support is a key regulator of neural-network formation and function [34,35,36,37]. It has been shown that deviations (i) in the formation of mitochondrial apparatus during the early period [that are able to delay the formation of interneuron connections] and (ii) in their efficiency delay brain maturation in OXYS rats and thus determine predisposition to the subsequent development of AD signs [38,39,40]. Analysis of transcriptome data from the hippocampus and prefrontal cortex during brain maturation have confirmed the decrease in the efficiency of neural-connection formation, and this problem is presumably explained mainly by a deficit in mitochondrial function. It is striking that the largest and most comparable differences in gene expression and in related processes have been observed in the early postnatal period and at the severe stage of the pathology [41].

Therefore, we hypothesized that genetic predisposition to accelerated aging and neurodegeneration in OXYS rats may be associated with early-life alterations in MAPK signaling during critical development of the brain, and that they evolve into the progressive pathway dysregulation observed during the development of the AD-like pathology. Here, we conducted parallel transcriptomic and proteomic analyses of MAPK cascades in the hippocampus and prefrontal cortex (PFC) of OXYS rats at P3 and P10 (time points of key brain developmental transitions) to identify the earliest molecular alterations that can serve as predictors of age-related neurodegeneration.

## 2. Results

### 2.1. Differential Expression of MAPK Signaling Pathway Genes in the Hippocampus and PFC of Wistar and OXYS Rats During the Early Postnatal Period

#### 2.1.1. Dynamics of Gene Expression of the p38 MAPK Signaling Pathway During Brain Development

**Figure 1:**
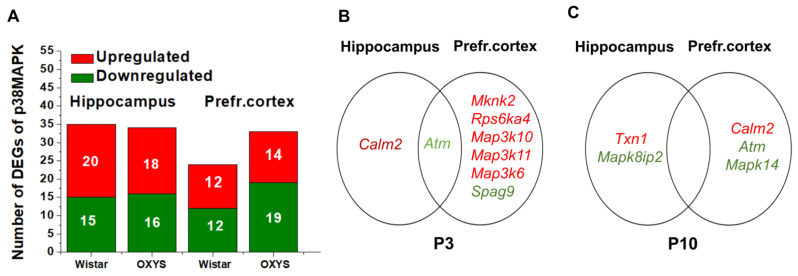
Dynamics of p38 MAPK signaling pathway gene expression during brain development. Age-related changes in the set of DEGs (defined as differences between ages 3 and 10 days) belonging to the p38 MAPK signaling pathway in the hippocampus and PFC of Wistar and OXYS rats (**A**). Venn diagrams for DEGs (defined as differences of OXYS rats from Wistar rats) in the hippocampus and PFC at the age of 3 days (**B**) and 10 days (**C**).

In the hippocampus, among 56 genes of the p38 MAPK signaling pathway, numbers of differentially expressed genes (DEGs) between P3 and P10 were similar when the two strains were compared (35 in Wistar rats vs. 34 in OXYS rats, Figure 1A), thereby indicating a conserved developmental program. In contrast, the PFC showed strain differences: in Wistar rats, the expression of 24 genes was found to be altered, while in OXYS rats, the expression of 33 genes was altered, with predominance of downregulation, implying region-specific alteration (Supplementary Tables S1 and S2, Figure S1). Functional analysis linked these genes to axon guidance (*Cdc42*, *Rac1*, and *Map2k6*), neurotrophin signaling (*Calm1*, *Calm2*, *Camk2b*, *Cdc42*, *Map3k5*, *Mapk11*, *Mapk14*, *Mapkapk2*, *Rac1*, and *Rps6ka5*), oxidative stress response (*Dusp1*, *Map2k6*, and *Mapk11*), and metabolic regulation (*Rps6ka5*, *Map2k6*, and *Cdc42*).

If we define DEGs as differences in gene expression between OXYS and Wistar rats, then in the hippocampus, there were minimal numbers of DEGs at both P3 and P10 (two genes each). By contrast, the PFC showed early vulnerability, with seven DEGs at age P3 decreasing to three DEGs by P10 (Figure 1B,C, Supplementary Table S3). These findings mean that the early postnatal period is characterized by intense transcriptional dynamics of p38 MAPK-related genes, with OXYS rats exhibiting a broader transcriptional response specifically in the PFC, predominantly showing gene downregulation that precedes hippocampal changes.

#### 2.1.2. Dynamics of Gene Expression of the ERK1/2 Signaling Pathway During Brain Development

**Figure 2:**
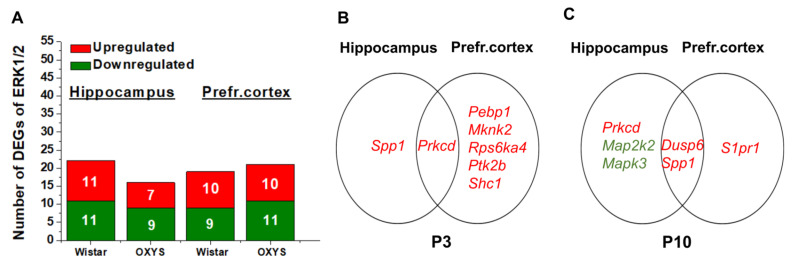
Dynamics of ERK1/2 signaling pathway gene expression during brain development. Age-related changes (DEGs are defined as differences between ages 3 and 10 days) of the ERK1/2 signaling pathway in the hippocampus and PFC of Wistar and OXYS rats (**A**). Venn diagrams for DEGs (defined as differences between the two strains) in the hippocampus and PFC at the age of 3 (**B**) and 10 (**C**) days.

When ages of 3 and 10 days were compared, in the hippocampus, there were 22 affected genes out of 54 genes of the ERK1/2 signaling pathway in Wistar rats and 16 in OXYS rats—a 27% reduction—thus pointing to a blunted transcriptional response in the OXYS hippocampus (Figure 2A). In the PFC, the pattern turned out to be reversed: Wistar rats showed changes in the expression of 19 genes versus 21 in OXYS rats (Figure 2A, (Supplementary Tables S1 and S2, Figure S1). Functional analysis linked these genes to neurotrophin signaling (*Braf*, *Hras*, *Kras*, *Map2k1*, *Map2k2*, *Mapk1*, *Mapk3*, *Nras*, *Prkcd*, and *Raf1*), synapse formation (*Braf*, *Hras*, *Map2k1*, *Mapk1*, *Mapk3*, *Prkcd*, *Ptk2b*, *Rps6ka1*, and *Rps6ka4*), homeostasis (*Kras*, *Mapk1*, *S1pr1*, and *Ptk2b*), and metabolic regulation (*Rps6ka5*, *Map2k6*, and *Cdc42*).

If we define DEGs as differences of OXYS rats from Wistar rats, then in the hippocampus at P3, there were minimal numbers DEGs (two genes), while the PFC showed six upregulated genes. At the age of P10, there were five hippocampal DEGs, and the set of PFC DEGs decreased to three (all upregulated; Figure 2B,C, Supplementary Table S3). Crucially, functional analysis revealed a dichotomy: upregulated genes in OXYS rats were associated with oxidative stress response (*Map2k1*, *Pebp1*, *Prkcd*, *Ptk2b*, and *Shc1*), synapse formation (*Map2k1*, *Pebp1*, *Prkcd*, *Ptk2b*, and *Rps6ka4*), and apoptosis (*Jun*, *Traf2*, and *Map3k5*), while the set of downregulated genes was found to be enriched with neurotrophin signaling *(Map2k1*, *Map2k2*, *Map3k1*, *Mapk3*, *Prkcd*, *Rps6ka3*, and *Rps6ka6*), mTOR signaling (*Map2k1*, *Map2k2*, *Mapk3*, *Rps6ka3*, and *Rps6ka6*), and long-term potentiation (*Map2k1*, *Map2k2*, *Mapk3*, *Rps6ka3*, and *Rps6ka6*). This imbalance—activation of stress pathways and suppression of adaptation genes—may cause susceptibility to an accelerated cognitive decline.

#### 2.1.3. Dynamics of Gene Expression of the JNK Signaling Pathway During Brain Development

**Figure 3:**
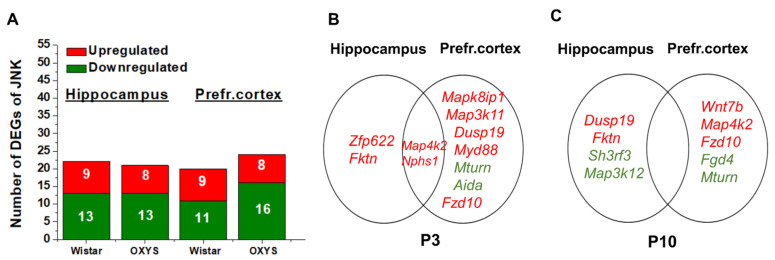
Dynamics of JNK signaling pathway gene expression during brain development. Age-related changes of the JNK signaling pathway (DEGs are defined as differences between ages 3 and 10 days) in the hippocampus and PFC of Wistar and OXYS rats (**A**). Venn diagrams for DEGs (defined as differences between the two strains) in the hippocampus and PFC at the age of 3 (**B**) and 10 (**C**) days.

To investigate JNK signaling in early brain development, we analyzed 33 pathway genes in the hippocampus and PFC of Wistar and OXYS rats at P3 and P10. Between these time points, hippocampal changes were similar between the two strains (22 DEGs in Wistar rats vs. 21 in OXYS rats, Figure 3A). In the PFC, OXYS rats showed more extensive changes (24 DEGs) than Wistar rats (20 DEGs), with marked predominance of downregulation (16 vs. 11 genes; Supplementary Tables S1 and S2, Figure S1). This suppression in the OXYS PFC was specific for the neurodegeneration genotype. Functional analysis linked these genes to stress response (*Hacd3*, *Mapk10*, *Mapk8ip1*, and *Map2k4*) and apoptosis (*Map2k4*, *Mapk10*, and *Map2k7*).

Among all MAPK cascades, we found the most extensive transcriptional differences between OXYS and Wistar rats in the JNK pathway (DEGs are now defined as differences between the two strains). At P3, hippocampal differences involved four genes (all upregulated), while the PFC showed nine DEGs (seven upregulated and two downregulated). By P10, hippocampal differences remained at four DEGs, and PFC differences consisted of five DEGs (Figure 3B,C).

### 2.2. Differential Activation of MAPKs in the Hippocampus and PFC of Wistar and OXYS Rats in the Early Postnatal Period

To determine whether transcriptional differences translated into functional alterations at the protein level, we quantified total and phosphorylated ERK1/2, p38 MAPK, and JNK1/2.

#### 2.2.1. Activation of p38 MAPK in the Hippocampus of Wistar and OXYS Rats at P3 and P10

**Figure 4:**
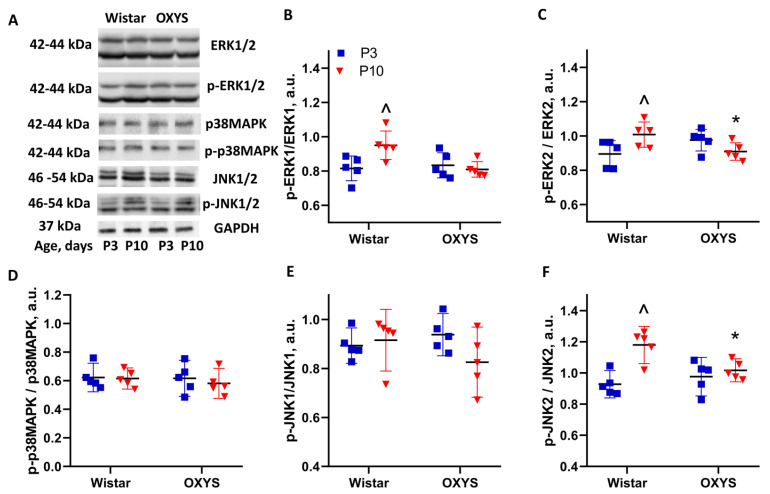
Changes in the levels of p38 MAPK, ERK1/2, and JNK1/2 phosphorylation in the hippocampus of Wistar and OXYS rats during early postnatal development according to Western blot analysis (**A**). Graphical representation of the ratio of p-ERK1 to ERK1 (**B**), p-ERK2 to ERK2 (**C**), p-p38 MAPK to p38 MAPK (**D**), p-JNK1 to JNK1 (**E**), p-JNK2 to JNK2 (**F**), The protein amounts were normalized to GAPDH. Mean ± SEM (n = 5). * Differences between Wistar and OXYS rats of the same age; ^ differences from rats of the same strain at a different age (*p* < 0.05).

Total p38 MAPK, phospho-p38 MAPK (p-p38 MAPK), and the phosphorylation ratio showed no age-related changes and no differences between the strains (Figure 4). p38 MAPK signaling at the protein level appeared remarkably stable during this developmental period, thus implying tight homeostatic control.

#### 2.2.2. Activation of ERK1/2 in the Hippocampus of Wistar and OXYS Rats at P3 and P10

Total ERK1 and ERK2 levels were age-dependent (F_1,16_ = 21.1, *p* < 0.001, and F_1,16_ = 11.4, *p* < 0.004, respectively), increasing in Wistar rats from P3 to P10 (*p* < 0.004), while OXYS rats showed no significant changes (Figure 4). p-ERK1 levels were age-dependent (F_1,16_ = 18.9, *p* < 0.001), increasing only in Wistar rats by day 10 (*p* < 0.001). On day 3, the p-ERK1 amount was greater in OXYS rats (*p* < 0.002), but by day 10, it fell below Wistar levels (*p* < 0.003). The phosphorylation ratio (p-ERK1/ERK1) increased in Wistar rats from P3 to P10 (*p* < 0.025) and was significantly lower in OXYS rats at P10 (*p* < 0.010; Figure 4). The p-ERK2 level was age-dependent (F_1,16_ = 11.3, *p* < 0.006), increasing in Wistar rats (*p* < 0.043) and resulting in a lower p-ERK2 amount in OXYS rats on day 10 (*p* < 0.043). The phosphorylation ratio increased in Wistar rats by day 10 (*p* < 0.048) and was lower in OXYS rats at this age (*p* < 0.039; Figure 4). These findings indicated a failure of OXYS rats to undergo the normal developmental increase in hippocampal ERK signaling; this problem likely led to reduced signaling capacity by age P10: a critical period for hippocampal-circuit formation.

#### 2.2.3. Activation of JNK in the Hippocampus of Wistar and OXYS Rats at P3 and P10

Total JNK1 and JNK2 levels depended on the genotype (i.e., strain; F_1,16_ = 41.7, *p* < 0.004, and F_1,16_ = 32.8, *p* < 0.001, respectively). In Wistar rats, JNK1 and JNK2 amounts increased dramatically by day 10 (*p* < 0.001 and *p* < 0.002, respectively) and became significantly greater than those in OXYS rats (*p* < 0.001; Figure 4). The amount of p-JNK1 increased with age in both strains (*p* < 0.001) without interstrain differences. The JNK1 phosphorylation ratio showed no changes. The p-JNK2 level increased with age in both strains (Wistar: *p* < 0.050; OXYS: *p* < 0.001) without interstrain differences. Of note, the JNK2 phosphorylation ratio increased in Wistar rats by day 10 (*p* < 0.002) and was significantly higher than that in OXYS rats (*p* < 0.012; Figure 4). A hippocampal finding was the complete failure of OXYS rats to increase JNK2 levels between P3 and P10, in stark contrast to the robust increases seen in Wistar rats. This developmental arrest in JNK accumulation, combined with reduced JNK2 phosphorylation at P10, indicates both quantitative and qualitative impairments in hippocampal JNK signaling in the OXYS strain.

#### 2.2.4. Activation of p38 MAPK in the PFC of Wistar and OXYS Rats at P3 and P10

**Figure 5:**
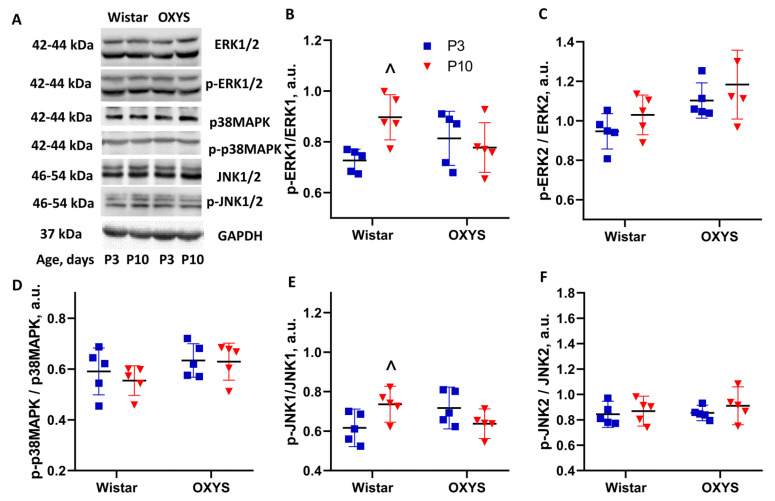
Changes in levels of p38 MAPK, ERK1/2, and JNK1/2 phosphorylation in the PFC of Wistar and OXYS rats during early postnatal development according to Western blot analysis (**A**). Graphical representation of the ratio of p-ERK1 to ERK1 (**B**), p-ERK2 to ERK2 (**C**), p-p38 MAPK to p38 MAPK (**D**), p-JNK1 to JNK1 (**E**), p-JNK2 to JNK2 (**F**), The protein amounts were normalized to GAPDH. Mean ± SEM (n = 5). ^ Differences from rats of the same strain at a different age (*p* < 0.05).

Just as in the hippocampus, in the PFC no differences in total p38 MAPK, p-p38 MAPK, or phosphorylation ratio were observed (Figure 5). p38 MAPK signaling proved to be stable among strains and regions during this developmental period.

#### 2.2.5. Activation of ERK1/2 in the PFC of Wistar and OXYS Rats at P3 and P10

Unlike the hippocampus, the PFC showed no differences between Wistar and OXYS rats in total ERK1, p-ERK1, or the ERK1 phosphorylation ratio (Figure 5). Nevertheless, the p-ERK1 level rose in Wistar rats by day 10 (*p* < 0.046), thereby leading to an increased phosphorylation ratio (*p* < 0.005). Total ERK2, p-ERK2, and ERK2 phosphorylation ratio showed no age-related changes and no interstrain differences (Figure 5). Thus, prefrontal ERK signaling appears to be largely preserved in OXYS rats, thereby highlighting region-specific vulnerability of the hippocampal ERK pathway.

#### 2.2.6. Activation of JNK in the PFC of Wistar and OXYS Rats at P3 and P10

Total JNK1 and JNK2 levels depended on the genotype (F_1,16_ = 8.9, *p* < 0.009, and F_1,16_ = 5.7, *p* < 0.029, respectively). In Wistar rats, the JNK1 amount increased by P10 (*p* < 0.010) and became significantly higher than that in OXYS rats (*p* < 0.003; Figure 5). The level of JNK2 was higher in OXYS rats at P3 (*p* < 0.032). p-JNK1 showed no changes. The JNK1 phosphorylation ratio went up in Wistar rats by P10 (*p* < 0.036) and was higher than that in OXYS rats (*p* < 0.049; Figure 5). p-JNK2 was influenced by both the genotype (F_1,16_ > = 4.7, *p* < 0.047) and age (F_1,16_ = 10.6, *p* < 0.005). At P3, the p-JNK2 amount was larger in OXYS rats (*p* < 0.019), but this difference disappeared by P10 owing to an increase seen in Wistar rats (*p* < 0.012). The JNK2 phosphorylation ratio manifested no differences (Figure 5). Consequently, the PFC of OXYS rats showed a JNK1 accumulation deficit similar to that observed in the hippocampus, with a significantly lower total JNK1 level and reduced JNK1 phosphorylation by day P10. JNK2 showed a distinct pattern: transient elevation at P3 that went back to normal by P10.

## 3. Discussion

There is growing evidence that the formation of aberrant neural networks during final stages of brain maturation in the early postnatal period, under the influence of genetic and/or environmental factors, may have long-term consequences and contribute to the development of sporadic AD. We have previously identified a delay in brain maturation in OXYS rats during a glial-support decrease promoting a reduction in the efficiency of interneuron contact formation. In this study, we performed an integrated analysis of MAPK pathways’ (ERK1/2, JNK, and p38 MAPK) activity in the hippocampus and PFC at key brain postnatal developmental transitions in healthy Wistar rats and in senescence-accelerated OXYS rats. Our findings show that delayed brain maturation in OXYS rats occurs simultaneously with alterations in MAPK signaling. These aberrations potentially are able to contribute to a decrease in the efficiency of neuronal contact formation and to the increased brain susceptibility to neurodegeneration later in life.

The period between P3 and P10 in rodents is critical for synaptogenesis, neuronal-network formation, and gliogenesis [15,35,36,37,42,43,44]. We recently found that at birth, the synaptic population in the PFC of OXYS rats is half of that in Wistar rats, and there is a delay in the formation of interneuronal connections and in their efficiency in the OXYS strain at age P0–P20 [38,39,40,41]. Here, we for the first time show that postnatal brain development is accompanied by extensive remodeling of gene expression in all three major MAPK pathways in both the hippocampus and PFC of healthy Wistar rats. This observation underscores the fundamental role of MAPKs signaling not only in stress responses but also in normal developmental programs [9,10,11,12,13,14,15,16,17,18,19,20,21,22,23,24]. In OXYS rats, however, the transcriptional dynamics of MAPK pathway genes were found to be altered. The most pronounced differences between the strains were observed in the PFC at P3, where the number of DEGs (defined as differences between the two strains) was maximal across all three MAPK pathways. Notably, by P10, these differences proved to be largely attenuated; as we have demonstrated previously, by P20 (during the period of completion of brain maturation), the activity of MAPK signaling pathways at the level of post-translational modifications in OXYS rats does not differ from that in control animals [31,32,33].

The most striking divergence was observed in the JNK pathway, with OXYS rats exhibiting the largest number of DEGs (defined as differences between P3 and P10) in both the hippocampus and PFC. The JNK pathway is a critical regulator of both developmental processes and stress responses [9,18,19,20,45,46]. Its dual role as a mediator of neuronal survival and apoptosis makes its regulation crucial during brain maturation. In addition, JNK is a central actor in the mechanisms of synapse degeneration that characterize both neurodevelopmental and neurodegenerative diseases [28,45]. The transcriptional changes we observed here, particularly the downregulation of JNK pathway components in the OXYS PFC, point to a fundamental shift in the balance between these opposing functions. This finding is consistent with our previous work indicating that the development and progression of the AD-like pathology in OXYS rats occur during JNK activation [33]. It is plausible that the early-life suppression of JNK signaling components creates a state of “latent vulnerability,” where the system is ill-equipped to handle later-life stressors, leading to the pathological overactivation observed in the aged animals.

The observed transcriptional changes only partially matched our protein level findings, thereby highlighting the importance of post-transcriptional and post-translational mechanisms in the regulation of MAPK cascades within the developing brain. JNKs play a dual role in development: they are essential for cytoskeletal dynamics, axonal guidance, and neuronal migration [17,18,19,20], but their chronic activation can promote cell death [16,25,28,29,45]. In OXYS rats, we observed not hyperactivation but rather a protein deficit, which may point to alteration of translational regulation or of protein stability. The reduced JNK1 levels in the PFC were accompanied by diminished relative phosphorylation by day P10, and this diminution further dampened signaling capacity. Given the role of JNK-dependent phosphorylation in the regulation of genes involved in synaptic function, this deficit could impair the formation of stable cortical networks [22,23,24].

Transcriptional remodeling of ERK pathway genes was also evident during the P3–P10 window. The most pronounced differences between the strains were observed in the PFC at P3, where the number of DEGs (defined as differences from the controls) was the greatest. Notably, by day P10, these differences largely got attenuated, and at P20—a stage corresponding to the completion of major brain maturation processes—the activity of this MAPK signaling pathway at the level of post-translational modifications does not differ between the two strains [31,32,33].

Despite these transcriptional differences within the PFC, the most significant alterations of ERK signaling at the protein level were confined to the hippocampus, thereby highlighting the differences in region-specific post-transcriptional regulation. Wistar rats displayed the expected age-related increase in the total ERK1/2 amount and in the level of the phosphorylated forms by P10; these observations reflect ongoing growth and synaptogenesis [14,15,22,47]. OXYS rats failed to show this developmental increase. In addition, p-ERK1 levels were below the control levels by P10, indicating a failure to sustain normal signaling throughout this critical period. Given the well-established role of ERK1/2 in memory consolidation and in long-term potentiation [21,22,23,24,47], this hippocampal deficit may be a molecular cause of the early cognitive decline characteristic of OXYS rats [30].

Transcriptional changes in p38 MAPK pathway genes were present but less pronounced than those observed for JNK and ERK. In contrast to the other two pathways, the expression and phosphorylation of p38 MAPK in both brain regions and in both strains were unchanged between P3 and P10. This absence of age-related differences contrasts with the dynamic p38 regulation reported by Costa et al. [48], who found phosphorylation peaks at P4 and sustained elevation starting from P10; these phenomena may be associated with peaks of apoptosis waves at these ages. This discrepancy may be explained by the broader age range examined in their study. Nevertheless, the lack of age-related differences in our study suggests that p38 MAPK is under tight homeostatic control during this specific developmental window. Its activation appears to be related to later stages of neurodegeneration, as we have previously demonstrated in adult OXYS rats [31], rather than a contributor to the initial predisposition to AD.

By the age of P20, the brain maturation process is largely completed, including the establishment of mature synaptic circuits and the consolidation of neuronal networks [15,42,43,44]. At this age, no significant differences in either the expression or phosphorylation levels of ERK1/2, JNK1/2, or p38 MAPK are detectable between OXYS and Wistar rats in either the hippocampus or PFC [31,32,33]. The apparent “recovery” of normal MAPK signaling by P20 may represent a compensatory response or the establishment of alternative regulatory mechanisms that temporarily restore pathway homeostasis. On the other hand, the early-life alterations occurring during a period of circuit formation may set the stage for the subsequent emergence of the AD-like pathology.

Thus, delayed brain maturation in OXYS rats occurs simultaneously with a shift in the activity of MAPK signaling pathways. These early alterations do not cause an immediate pathology but, consistently with the Developmental Origins of Health and Disease concept [1,3,4,5], establish a phenotype of heightened vulnerability. The normalization of MAPK signaling by P20, coinciding with the completion of brain maturation, suggests that the critical period for these developmental impairments is confined to the first 2 postnatal weeks. Nonetheless, the transient nature of these molecular abnormalities does not diminish their pathological significance; rather, this transient nature indicates that even temporary perturbations during sensitive developmental windows can have lasting consequences for brain health, thereby predisposing them to age-related neurodegeneration long after the initial insult has resolved.

## 4. Materials and Methods

### 4.1. Animals

Male Wistar and OXYS rats were obtained from the breeding colony at the Institute of Cytology and Genetics, the Siberian Branch of the Russian Academy of Sciences (Novosibirsk, Russia). Rats were housed under standard conditions (12 h light/dark cycle, 22 ± 2 °C, 60% relative humidity) with *ad libitum* access to standard rodent chow and water. Every effort was made to minimize the number of animals used and their discomfort. Rats were euthanized by decapitation on days P3 and P10 (n = 5 per age and strain). Brains were rapidly dissected; the hippocampus and PFC (n = 5 per age and strain) were isolated, immediately frozen in liquid nitrogen, and stored at −80 °C until Western blot analysis. The PFC and hippocampus (n = 3) were placed in RNALater (Ambion, Austin, TX, USA, catalog # AM7020), frozen, and stored at −20 °C until RNA-seq.

### 4.2. RNA Extraction

Hippocampi and PFCs were collected from Wistar rats (controls) and OXYS rats aged 3 and 10 days (n = 4). More than 40 million single-end reads 50 bp long were obtained for each sample of cortical RNA, by Illumina nonstranded sequencing on an Illumina GAIIx instrument at the Genoanalitika Lab (Moscow, Russia) [http://www.genoanalytica.ru/, (accessed on 1 September 2021)] in accordance with standard Illumina protocols (mRNA-Seq Sample Prep Kit). Briefly, mRNA was isolated from total RNA using Sera-Mag Magnetic Oligo (dT; Cytiva, Fremont, CA, USA) beads and then broken into small fragments by means of divalent cations and heating. Using a reverse transcriptase and random primers, we synthesized first- and second-strand cDNAs. Each cDNA was processed in an end repair reaction with T4 DNA polymerase and Klenow DNA polymerase to blunt the termini. An “A” base was then added to the 3′ end of the blunt phosphorylated DNA fragments, and an Illumina adaptor with a single T overhang at its 3′ end was next ligated to the end of the DNA fragment, for hybridization in a single-read flow cell. After that, a size range of cDNA templates was selected, and these fragments were amplified on a cluster station using Single-Read Cluster Generation Kit v2 (Illumina, Hayward, CA, USA). Sequencing-by-synthesis (SBS) at 50-nucleotide length was performed by means of SBS v4 reagents on Genome Analyzer IIx running the SCS2.8 software (Illumina).

### 4.3. Functional Enrichment Analysis

To identify the Gene Ontology (GO) terms associated with p38 MAPK signaling over-represented in a DEG list, the detected DEGs were employed for functional enrichment analyses with the help of the DAVID tool (Database for Annotation, Visualization and Integrated Discovery, https://davidbioinformatics.nih.gov/ (accessed on 1 December 2025)). Pathway analysis of the DEGs associated with the MAPK signaling pathways (Rat Genome Database, https://rgd.mcw.edu/ (accessed on 1 December 2025)) was carried out via the Kyoto Encyclopedia of Genes and Genomes (KEGG) pathways (http://www.genome.jp/kegg/ (accessed on 1 December 2025)).

### 4.4. Protein Extraction and Western Blot Analysis

Hippocampi and PFCs from Wistar (control) and OXYS rats aged 3 and 10 days (n = 5) were homogenized in RIPA buffer (50 mM Tris-HCl pH 7.4, 150 mM NaCl, 1% of Triton X-100, 1% of sodium deoxycholate, 0.1% of sodium dodecyl sulfate, and 1 mM ethylenediaminetetraacetic acid) containing protease and phosphatase inhibitor cocktails (Sigma-Aldrich, St. Louis, MI, USA). The homogenates were centrifuged at 12,000× *g* for 30 min at 4 °C, and supernatants were collected as the total-protein fraction. Protein concentration was determined using the BCA assay (Thermo Scientific, Rockford, IL, USA). Proteins were separated by electrophoresis in a 12% polyacrylamide gel, transferred to a nitrocellulose membrane (Bio-Rad, Hercules, CA, USA), and blocked with 5% bovine serum albumin in Tris-buffered saline containing 0.1% of Tween 20 (TBST) for 1 h. Next, the membrane was incubated at 4 °C overnight with primary antibodies against p38 MAPK, phospho-p38 MAPK (Thr180, Tyr182), ERK1/ERK2, phospho-ERK1/ERK2 (Thr202, Tyr204), JNK1/2, phospho-JNK1/2, JNK3, phospho-JNK3 (# 33-1300, # 36-8500, # 36-8800, # 13-6200, AHO1362, 44-682G, PA5-36753, MA5-31795, respectively; dilution 1:1000; Invitrogen, Eugene, OR, USA), and β-actin as a loading control and then for 1 h with secondary antibodies (anti-rabbit and anti-mouse: ab6721 and ab97046; Abcam, Boston, MA, USA) dilution 1:5000). Signals were visualized using the ECL substrate (Bio-Rad, Hercules, CA, USA). The intensity of luminescence was evaluated with the help of a ChemiDoc MP imaging system (Bio-Rad) and ImageJ software, version 7.12 (NIH, Bethesda, MD, USA).

### 4.5. Statistical Analysis

The Newman–Keuls post hoc test in STATISTICA 8.0 software (StatSoft, Tulsa, OK, USA) was applied to significant main effects and interactions in order to evaluate differences between some sets of means. One-way ANOVA was performed for pairwise group comparisons. For Western-blot data, two-way ANOVA with factors “genotype” (Wistar or OXYS) and “age” (day 3 or day 10) was employed. The data are presented as mean ± SEM. Differences were regarded as statistically significant at *p* < 0.05.

## 5. Conclusions

This study indicates that genetic predisposition to accelerated senescence and neurodegeneration in OXYS rats may be associated with early-life alterations in MAPK signaling pathways. Extensive transcriptional remodeling of p38, ERK, and JNK pathway genes was noted during early postnatal brain development, with OXYS rats showing distinct patterns, particularly in the PFC. Strain-specific transcriptional changes involved genes critical for neurodevelopment, neurotrophin signaling, oxidative stress response, and synaptic plasticity. Protein level deficits in OXYS rats (including impaired age-dependent accumulation of JNK1/2 in the hippocampus and PFC and an insufficient increase in ERK1/2 and JNK2 phosphorylation within the hippocampus and in JNK1 phosphorylation within the PFC) were registered by P10. Preserved p38 MAPK protein activation, despite the transcriptional changes, is suggestive of pathway-specific regulation during development.

These findings provide evidence that the molecular basis of age-related neurodegeneration may be established during early brain development, long before clinical manifestations.

## Data Availability

The original data presented in this study are found in the article/Supplementary Material. Further inquiries can be directed to the corresponding author.

## Supplementary Materials

The following supporting information can be downloaded at: https://www.mdpi.com/article/10.3390/ijms27125430/s1.
