# Supplementary material for: Features of Alteration in MAPK Pathway Activity in the Postnatal Brain of a Rat Model of Sporadic Alzheimer’s Disease
Source: Int J Mol Sci. 2026 Jun 16;27(12):5430. doi: 10.3390/ijms27125430 (PMC13299555; doi:10.3390/ijms27125430)
Supplement: Supplementary file 1 [file ijms-27-05430-s001.zip › Supplementary Figure S1.pdf]

Supplementary Figure S1.

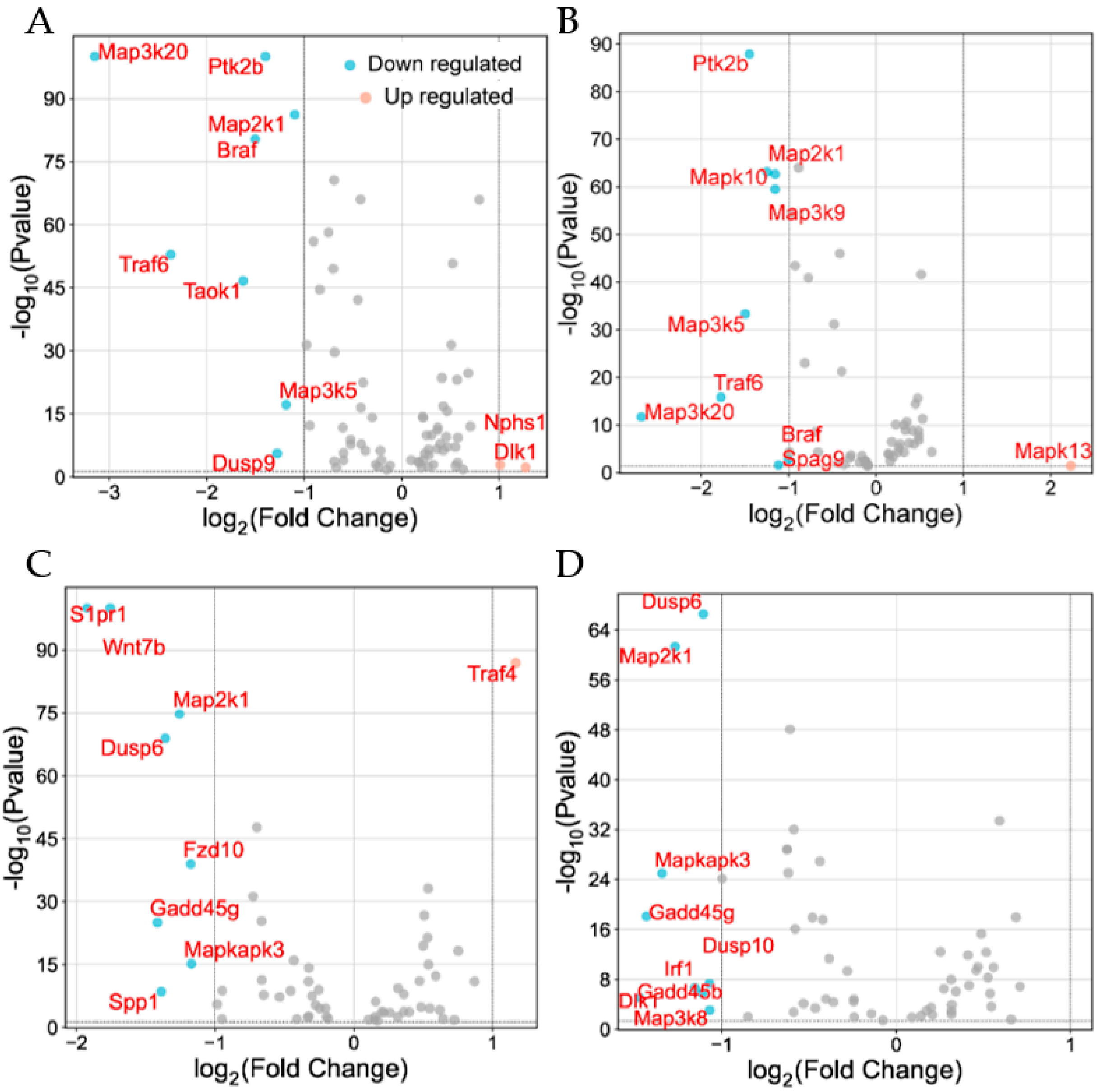

Figure S1. Age-related changes of the MAPK signaling pathways (DEGs defined as differences between ages 3 and 10 days) in hippocampus of Wistar (A) and OXYS rats (B), and in prefrontal cortex rats of Wistar (C) and OXYS rats (D) are shown in a volcano plot.
