# Supplementary material for: Features of Alteration in MAPK Pathway Activity in the Postnatal Brain of a Rat Model of Sporadic Alzheimer’s Disease
Source: Int J Mol Sci. 2026 Jun 16;27(12):5430. doi: 10.3390/ijms27125430 (PMC13299555; doi:10.3390/ijms27125430)
Supplement: Supplementary file 1 [file ijms-27-05430-s001.zip › Supplementary Tables.pdf]

Supplementary Table S1. The list of genes involved in MAPK signaling pathways (ERK1/2, JNK, and p38), the expression of which changes in the hippocampus of Wistar and OXYS rats from age P3 to P10.

Gene ID	Gene symbol	Gene name	MAPK pathway	log2Fold Change	padj
**Wistar from age P3 to P10**					
ENSRNOG00000012081	Txn1	thioredoxin 1	p38	-0,22	1,90E-06
ENSRNOG00000029773	Atm	ATM serine/threonine kinase	p38	-0,27	2,83E-03
ENSRNOG00000011789	Cdon	cell adhesion molecule-related/down-regulated by oncogenes	p38	-0,37	1,83E-06
ENSRNOG00000016770	Calm2	calmodulin 2	p38	-0,40	2,66E-22
ENSRNOG00000010381	Mknk1	MAPK interacting serine/threonine kinase 1	p38; ERK1/2	-0,42	5,83E-08
ENSRNOG00000004060	Calm1	calmodulin 1	p38	-0,42	2,32E-65
ENSRNOG00000004005	Camk2b	calcium/calmodulin-dependent protein kinase II beta	p38	-0,45	1,09E-41
ENSRNOG00000014832	Mapkapk3	MAPK activated protein kinase 3	p38	-0,60	6,37E-06
ENSRNOG00000019822	Gadd45b	growth arrest and DNA-damage-inducible, beta	p38; JNK	-0,69	5,61E-04
ENSRNOG00000002749	Spag9	sperm associated antigen 9	p38	-0,70	4,96E-49
ENSRNOG00000012568	Map3k5	mitogen-activated protein kinase kinase kinase 5	p38	-1,18	3,91E-17
ENSRNOG00000055185	Dusp9	dual specificity phosphatase 9	p38	-1,28	8,56E-06
ENSRNOG00000015692	Taok1	TAO kinase 1	p38	-1,63	3,59E-46
ENSRNOG00000004639	Traf6	TNF receptor associated factor 6	p38	-2,37	2,14E-52
ENSRNOG00000017388	Map3k20	mitogen-activated protein kinase kinase kinase 20	p38	-3,15	8,86E-104
ENSRNOG00000019584	Dlk1	delta like non-canonical Notch ligand 1	p38	1,27	1,19E-02
ENSRNOG00000023945	Dusp10	dual specificity phosphatase 10	p38; JNK	0,57	1,09E-03
ENSRNOG00000003977	Dusp1	dual specificity phosphatase 1	p38; JNK	0,55	2,69E-07
ENSRNOG00000004362	Rps6ka5	ribosomal protein S6 kinase A5	p38; ERK1/2	0,47	2,62E-07
ENSRNOG00000006984	Mapk11	mitogen-activated protein kinase 11	p38	0,47	1,24E-15
ENSRNOG00000002659	Bnip2	BCL2/adenovirus E1B interacting protein 2	p38	0,42	1,67E-06
ENSRNOG00000029028	Mknk2	MAPK interacting serine/threonine kinase 2	p38; ERK1/2	0,41	2,20E-23
ENSRNOG00000004726	Mapkapk2	MAPK activated protein kinase 2	p38	0,37	8,05E-12

ENSRNOG00000001138	Taok3	TAO kinase 3	p38	0,37	3,38E-11
ENSRNOG00000003329	Ppm1d	protein phosphatase, Mg2+/Mn2+ dependent, 1D	p38	0,32	3,08E-03
ENSRNOG00000004437	Map2k6	mitogen-activated protein kinase kinase 6	p38	0,28	1,84E-05
ENSRNOG00000001345	Mapkapk5	MAPK activated protein kinase 5	p38	0,28	9,62E-07
ENSRNOG00000031875	Tab2	TGF-beta activated kinase 1/MAP3K7 binding protein 2	p38	0,26	5,60E-10
ENSRNOG00000063827	Tab1	TGF-beta activated kinase 1/MAP3K7 binding protein 1	p38	0,24	9,02E-06
ENSRNOG00000019964	Taok2	TAO kinase 2	p38	0,22	4,37E-14
ENSRNOG00000013536	Cdc42	cell division cycle 42	p38; JNK	0,21	2,53E-14
ENSRNOG00000009191	Ccm2	cerebral cavernous malformation 2	p38	0,21	3,17E-04
ENSRNOG00000021117	Rps6ka4	ribosomal protein S6 kinase A4	p38; ERK1/2	0,20	1,28E-02
ENSRNOG00000000513	Mapk14	mitogen-activated protein kinase 14	p38	0,18	1,22E-03
ENSRNOG00000025911	Rac1	Rac family small GTPase 1	p38	0,10	2,71E-04
ENSRNOG00000005175	Spp1	sphingosine-1-phosphate phosphatase 1	ERK1/2	0,63	3,41E-02
ENSRNOG00000042411	Rps6ka1	ribosomal protein S6 kinase A1	ERK1/2	0,57	1,87E-09
ENSRNOG00000018483	Smad1	SMAD family member 1	ERK1/2	0,57	5,85E-23
ENSRNOG00000010153	Raf1	Raf-1 proto-oncogene, serine/threonine kinase	ERK1/2	0,52	2,85E-50
ENSRNOG00000020005	Map2k2	mitogen-activated protein kinase kinase 2	ERK1/2	0,51	3,83E-31
ENSRNOG00000002592	Rps6ka6	ribosomal protein S6 kinase A6	ERK1/2	0,46	1,27E-09
ENSRNOG00000016346	Prkcd	protein kinase C delta	ERK1/2	0,27	8,72E-03
ENSRNOG00000053583	Mapk3	mitogen-activated protein kinase 3	ERK1/2	0,17	6,14E-04
ENSRNOG00000023079	Nras	GTPase NRas precursor (Transforming protein N-Ras)	ERK1/2	-0,21	2,99E-04
ENSRNOG00000023896	Dusp6	dual specificity phosphatase 6	ERK1/2	-0,24	9,96E-03
ENSRNOG00000009338	Kras	GTPase KRas precursor (K-Ras 2)	ERK1/2	-0,53	6,35E-09
ENSRNOG00000012818	Ksr1	kinase suppressor of ras 1	ERK1/2	-0,61	9,09E-12
ENSRNOG00000014089	Map3k2	mitogen-activated protein kinase kinase kinase 2	ERK1/2	-0,67	1,26E-02
ENSRNOG00000006890	Mapk1	mitogen-activated protein kinase 1	ERK1/2	-0,75	1,36E-57

ENSRNOG00000013683	S1pr1	sphingosine-1-phosphate receptor 1	ERK1/2	-0,84	4,04E-44
ENSRNOG00000021184	Map2k1	mitogen-activated protein kinase kinase 1	ERK1/2	-1,10	2,29E-85
ENSRNOG00000026853	Ptk2b	protein tyrosine kinase 2 beta	ERK1/2	-1,40	2,80E-142
ENSRNOG00000010957	Braf	B-Raf proto-oncogene, serine/threonine kinase	ERK1/2	-1,50	1,47E-79
ENSRNOG00000053200	Nphs1	nephrin	JNK	1,01	3,32E-03
ENSRNOG00000013169	Traf4	TNF receptor associated factor 4	JNK	0,79	2,56E-65
ENSRNOG00000010358	Sh3rf3	SH3 domain containing ring finger 3	JNK	0,70	4,97E-12
ENSRNOG00000048782	Wnt7a	Wnt family member 7A	JNK	0,68	1,71E-24
ENSRNOG00000000018	Cdc42ep5	CDC42 effector protein 5	JNK	0,55	5,59E-03
ENSRNOG00000058805	Aida	axin interactor, dorsalization associated	JNK	0,43	8,82E-17
ENSRNOG00000007387	Per1	period circadian regulator 1	JNK	0,39	4,89E-08
ENSRNOG00000001047	Map2k7	mitogen-activated protein kinase kinase 7	JNK	0,36	7,73E-07
ENSRNOG00000010728	Stradb	STE20-related kinase adaptor beta	JNK	0,30	4,94E-10
ENSRNOG00000009562	Mapk8ip1	mitogen-activated protein kinase 8 interacting protein 1	JNK	-0,12	4,88E-03
ENSRNOG00000013612	Map4k2	mitogen-activated protein kinase kinase kinase kinase 2	JNK	-0,16	3,74E-02
ENSRNOG00000010697	Hacd3	very-long-chain (3R)-3-hydroxyacyl-CoA dehydratase 3	JNK	-0,30	3,92E-14
ENSRNOG00000010260	Dixdc1	DIX domain containing 1	JNK	-0,42	2,00E-16
ENSRNOG00000023226	Zfp622	zinc finger protein 622	JNK	-0,52	6,62E-08
ENSRNOG00000055647	Tpd52l1	TPD52 like 1	JNK	-0,60	2,97E-04
ENSRNOG00000002516	Mturn	maturin, neural progenitor differentiation regulator homolog	JNK	-0,69	2,07E-29
ENSRNOG00000028419	Fktn	fukutin	JNK	-0,69	6,96E-70
ENSRNOG00000003834	Map2k4	mitogen-activated protein kinase kinase 4	JNK	-0,90	1,80E-55
ENSRNOG00000001818	Fgd4	FYVE, RhoGEF and PH domain containing 4	JNK	-0,94	2,99E-12
ENSRNOG00000025423	Map3k13	mitogen-activated protein kinase kinase kinase 13	JNK	-0,98	3,99E-32
**OXYS from age P3 to P10**					
ENSRNOG00000010838	Araf	A-Raf proto-oncogene, serine/threonine kinase	ERK1/2	-0,14	6,91E-03
ENSRNOG00000010957	Braf	B-Raf proto-oncogene, serine/threonine kinase	ERK1/2	-1,01	4,26E-03

ENSRNOG00000004060	Calm1	calmodulin 1	p38	-0,42	2,52E-45
ENSRNOG00000016770	Calm2	calmodulin 2	p38	-0,48	1,02E-30
ENSRNOG00000004005	Camk2b	calcium/calmodulin-dependent protein kinase II beta	p38	-0,39	5,32E-21
ENSRNOG00000060825	Ccm2	CCM2 scaffold protein	p38	0,18	7,55E-07
ENSRNOG00000013536	Cdc42	cell division cycle 42	p38, JNK	0,22	3,90E-10
ENSRNOG00000003977	Dusp1	dual specificity phosphatase 1	p38, JNK	0,49	5,71E-07
ENSRNOG00000004003	Dusp10	dual specificity phosphatase 10	p38, JNK	0,64	1,28E-04
ENSRNOG00000006628	Dusp16	dual specificity phosphatase 16	p38, JNK	-0,37	3,54E-02
ENSRNOG00000055185	Dusp9	dual specificity phosphatase 9	p38	-0,99	1,42E-04
ENSRNOG00000019822	Gadd45b	growth arrest and DNA-damage-inducible, beta	p38	-0,67	1,51E-04
ENSRNOG00000016611	Hras	HRas proto-oncogene, GTPase	ERK1/2	-0,11	6,46E-02
ENSRNOG00000009338	Kras	KRAS proto-oncogene, GTPase	ERK1/2	-0,30	1,99E-03
ENSRNOG00000012818	Ksr1	kinase suppressor of ras 1	ERK1/2	-0,82	9,89E-23
ENSRNOG00000010176	Map2k1	mitogen activated protein kinase kinase 1	ERK1/2	-1,16	8,86E-62
ENSRNOG00000020005	Map2k2	mitogen activated protein kinase kinase 2	ERK1/2	0,35	1,12E-10
ENSRNOG00000003834	Map2k4	mitogen activated protein kinase kinase 4	JNK	-0,93	8,31E-43
ENSRNOG00000004437	Map2k6	mitogen-activated protein kinase kinase 6	p38	0,42	7,38E-09
ENSRNOG00000001047	Map2k7	mitogen activated protein kinase kinase 7	JNK	0,32	1,36E-04
ENSRNOG00000023521	Map3k10	mitogen activated protein kinase kinase kinase 10	p38, JNK	-0,11	3,78E-02
ENSRNOG00000001515	Map3k20	mitogen-activated protein kinase kinase kinase 20	p38	-2,69	1,09E-11
ENSRNOG00000031700	Map3k5	mitogen-activated protein kinase kinase kinase 5	p38	-1,50	7,55E-33
ENSRNOG00000007271	Map3k9	mitogen-activated protein kinase kinase kinase 9	JNK	-1,16	1,30E-58
ENSRNOG00000001849	Mapk1	mitogen activated protein kinase 1	ERK1/2	-0,78	2,69E-40
ENSRNOG00000002079	Mapk10	mitogen activated protein kinase 10	JNK	-1,25	3,35E-62
ENSRNOG00000006984	Mapk11	mitogen-activated protein kinase 11	p38	0,49	8,48E-08
ENSRNOG00000000515	Mapk13	mitogen activated protein kinase 13	p38	2,23	6,26E-02
ENSRNOG00000000513	Mapk14	mitogen activated protein kinase 14	p38	0,16	8,56E-03

ENSRNOG00000058478	Mapk8ip1	mitogen-activated protein kinase 8 interacting protein 1	JNK	-0,16	8,31E-04
ENSRNOG00000032828	Mapk8ip2	mitogen-activated protein kinase 8 interacting protein 2	p38, JNK	-0,11	2,12E-02
ENSRNOG00000033568	Mapk8ip3	mitogen-activated protein kinase 8 interacting protein 3	JNK	-0,12	7,86E-03
ENSRNOG00000002823	Mapk9	mitogen-activated protein kinase 9	JNK	-0,89	4,33E-63
ENSRNOG00000004726	Mapkapk2	MAPK activated protein kinase 2	p38	0,53	2,58E-11
ENSRNOG00000014832	Mapkapk3	MAPK activated protein kinase 3	p38	-0,70	1,30E-08
ENSRNOG00000001345	Mapkapk5	MAPK activated protein kinase 5	p38	0,26	1,94E-05
ENSRNOG00000010381	Mknk1	MAPK interacting serine/threonine kinase 1	p38	-0,29	6,10E-04
ENSRNOG00000029028	Mknk2	MAPK interacting serine/threonine kinase 2	p38	0,34	6,26E-10
ENSRNOG00000003329	Ppm1d	protein phosphatase, Mg2+/Mn2+ dependent, 1D	p38	0,38	2,43E-06
ENSRNOG00000027839	Ptk2b	protein tyrosine kinase 2 beta	ERK1/2	-1,45	1,28E-86
ENSRNOG00000001068	Rac1	Rac family small GTPase 1	p38, JNK	0,14	3,11E-04
ENSRNOG00000010153	Raf1	Raf-1 proto-oncogene, serine/threonine kinase	ERK1/2	0,51	5,27E-41
ENSRNOG00000014316	Rapgef1	Rap guanine nucleotide exchange factor 1	JNK	0,25	1,21E-04
ENSRNOG00000042411	Rps6ka1	ribosomal protein S6 kinase A1	ERK1/2	0,43	3,51E-06
ENSRNOG00000013194	Rps6ka2	ribosomal protein S6 kinase A2	ERK1/2	-0,09	5,71E-02
ENSRNOG00000005271	Rps6ka5	ribosomal protein S6 kinase A5	p38	0,47	1,68E-15
ENSRNOG00000002592	Rps6ka6	ribosomal protein S6 kinase A6	ERK1/2	0,48	8,25E-09
ENSRNOG00000013683	S1pr1	sphingosine-1-phosphate receptor 1	ERK1/2	-0,96	8,76E-54
ENSRNOG00000018483	Smad1	SMAD family member 1	ERK1/2	0,45	2,39E-14
ENSRNOG00000002749	Spag9	sperm associated antigen 9	p38	-0,44	1,93E-02
ENSRNOG00000017285	Tab1	TGF-beta activated kinase 1/MAP3K7 binding protein 1	p38	0,27	3,48E-06
ENSRNOG00000016054	Tab2	TGF-beta activated kinase 1/MAP3K7 binding protein 2	p38	0,32	5,87E-11
ENSRNOG00000015692	Taok1	TAO kinase 1	p38	-1,12	4,52E-02

ENSRNOG00000019964	Taok2	TAO kinase 2	p38	0,15	3,81E-04
ENSRNOG00000001138	Taok3	TAO kinase 3	p38	0,20	1,01E-03
ENSRNOG00000004639	Traf6	TNF receptor associated factor 6	p38	-1,78	1,04E-15
ENSRNOG00000012081	Txn1	thioredoxin 1	p38	-0,19	1,48E-02

Supplementary Table S2. The list of genes involved in MAPK signaling pathways (ERK1/2, JNK, and p38), the expression of which changes in the PFC of Wistar and OXYS rats from age P3 to P10.

Gene ID	Gene symbol	Gene name	MAPK pathway	log2Fold Change	padj
**Wistar from age P3 to P10**					
ENSRNOG00000058805	Aida	axin interactor, dorsalization associated	JNK	0.360	5.20E-08
ENSRNOG00000020022	Araf	A-Raf proto-oncogene, serine/threonine kinase	ERK1/2	-0.277	1.34E-05
ENSRNOG00000002659	Bnip2	BCL2/adenovirus E1B interacting protein 2	p38	0.590	3.27E-12
ENSRNOG00000004060	Calm1	calmodulin 1	p38	-0.699	4.55E-47
ENSRNOG00000016770	Calm2	calmodulin 2	p38	-0.457	8.14E-09
ENSRNOG00000004005	Camk2b	calcium/calmodulin-dependent protein kinase II beta	p38	-0.433	7.16E-16
ENSRNOG00000013536	Cdc42	cell division cycle 42	p38	0.157	2.58E-06
ENSRNOG00000000018	Cdc42ep5	CDC42 effector protein 5	JNK	0.619	2.09E-04
ENSRNOG00000011789	Cdon	cell adhesion molecule-related/down-regulated by oncogenes	p38	0.398	5.57E-04
ENSRNOG00000019584	Dlk1	delta like non-canonical Notch ligand 1	p38	-0.952	2.02E-02
ENSRNOG00000010260	Dixdc1	DIX domain containing 1	JNK	0.531	3.62E-21
ENSRNOG00000023945	Dusp10	dual specificity phosphatase 10	p38	-0.986	1.02E-05
ENSRNOG00000023896	Dusp6	dual specificity phosphatase 6	ERK1/2	-1.360	4.69E-68
ENSRNOG00000001818	Fgd4	FYVE, RhoGEF and PH domain containing 4	JNK	0.869	5.65E-11
ENSRNOG00000028419	Fktn	fukutin	JNK	-0.246	9.44E-05
ENSRNOG00000022554	Fzd10	frizzled class receptor 10	JNK	-1.177	2.01E-38
ENSRNOG00000019821	Gadd45a	growth arrest and DNA-damage-inducible, alpha	p38	-0.333	9.17E-03
ENSRNOG00000051252	Gadd45g	growth arrest and DNA-damage-inducible, gamma	p38	-1.416	1.07E-24
ENSRNOG00000010697	Hacd3	very-long-chain (3R)-3-hydroxyacyl-CoA dehydratase 3	JNK	-0.253	4.80E-09
ENSRNOG00000019793	Hras	HRas proto-oncogene, GTPase	ERK1/2	-0.185	1.79E-02

ENSRNOG00000021184	Map2k1	mitogen-activated protein kinase kinase 1	ERK1/2	-1.257	8.20E-74
ENSRNOG00000020005	Map2k2	mitogen-activated protein kinase kinase 2	ERK1/2	0.318	2.13E-09
ENSRNOG00000003834	Map2k4	mitogen-activated protein kinase kinase 4	JNK	-0.666	4.73E-25
ENSRNOG00000004437	Map2k6	mitogen-activated protein kinase kinase 6	p38	0.260	8.05E-04
ENSRNOG00000001047	Map2k7	mitogen-activated protein kinase kinase 7	JNK	0.172	4.70E-02
ENSRNOG00000014428	Map3k10	mitogen-activated protein kinase kinase kinase 10	p38	-0.327	5.81E-11
ENSRNOG00000025423	Map3k13	mitogen-activated protein kinase kinase kinase 13	JNK	0.547	8.92E-05
ENSRNOG00000014089	Map3k2	mitogen-activated protein kinase kinase kinase 2	ERK1/2	0.723	3.22E-02
ENSRNOG00000012568	Map3k5	mitogen-activated protein kinase kinase kinase 5	p38	-0.664	2.76E-11
ENSRNOG00000006890	Mapk1	mitogen-activated protein kinase 1	ERK1/2	-0.727	8.90E-31
ENSRNOG00000003574	Mapk10	mitogen-activated protein kinase 10	JNK	-0.325	5.26E-03
ENSRNOG00000000513	Mapk14	mitogen-activated protein kinase 14	p38	0.151	2.94E-02
ENSRNOG00000009562	Mapk8ip1	mitogen-activated protein kinase 8 interacting protein 1	JNK	-0.325	3.86E-14
ENSRNOG00000014832	Mapkapk3	MAPK activated protein kinase 3	p38	-1.172	4.59E-15
ENSRNOG00000001345	Mapkapk5	MAPK activated protein kinase 5	p38	0.217	6.75E-04
ENSRNOG00000010381	Mknk1	MAPK interacting serine/threonine kinase 1	p38;ERK1/2	-0.536	2.16E-07
ENSRNOG00000029028	Mknk2	MAPK interacting serine/threonine kinase 2	p38;ERK1/2	0.507	2.37E-26
ENSRNOG00000002516	Mturn	maturin, neural progenitor differentiation regulator homolog	JNK	0.752	4.58E-18
ENSRNOG00000020713	Myd88	MYD88 innate immune signal transduction adaptor	JNK	-0.412	1.97E-02
ENSRNOG00000023079	Nras	GTPase NRas	ERK1/2	0.473	4.60E-11
ENSRNOG00000003329	Ppm1d	protein phosphatase, Mg^{2+}/Mn^{2+} dependent, 1D	p38	0.316	6.99E-03
ENSRNOG00000010153	Raf1	Raf-1 proto-oncogene, serine/threonine kinase	ERK1/2	0.535	1.04E-32
ENSRNOG00000042411	Rps6ka1	ribosomal protein S6 kinase A1	ERK1/2	0.359	4.88E-04
ENSRNOG00000007253	Rps6ka2	ribosomal protein S6 kinase A2	ERK1/2	-0.206	8.34E-05

ENSRNOG00000004362	Rps6ka5	ribosomal protein S6 kinase A5	p38;ERK1/2	0.538	7.07E-15
ENSRNOG00000002592	Rps6ka6	ribosomal protein S6 kinase A6	ERK1/2	0.484	4.49E-05
ENSRNOG00000013683	S1pr1	sphingosine-1-phosphate receptor 1	ERK1/2	-1.924	8.66E-266
ENSRNOG00000018483	Smad1	SMAD family member 1	ERK1/2	0.500	2.69E-19
ENSRNOG00000005175	Spp1	sphingosine-1-phosphate phosphatase 1	ERK1/2	-1.389	1.32E-08
ENSRNOG00000010728	Stradb	STE20-related kinase adaptor beta	JNK	0.151	7.21E-03
ENSRNOG00000031875	Tab2	TGF-beta activated kinase 1/MAP3K7 binding protein 2	p38	0.106	2.70E-02
ENSRNOG00000001138	Taok3	TAO kinase 3	p38	0.202	5.39E-04
ENSRNOG00000055647	Tpd52l1	TPD52 like 1	JNK	-0.950	7.32E-09
ENSRNOG00000013169	Traf4	TNF receptor associated factor 4	JNK	1.166	7.25E-86
ENSRNOG00000004639	Traf6	TNF receptor associated factor 6	p38	0.643	1.94E-02
ENSRNOG00000012081	Txn1	thioredoxin 1	p38	-0.302	1.25E-07
ENSRNOG00000048782	Wnt7a	Wnt family member 7A	JNK	-0.197	6.24E-03
ENSRNOG00000008745	Wnt7b	Wnt family member 7B	JNK	-1.757	1.07E-102
ENSRNOG00000023226	Zfp622	zinc finger protein 622	JNK	-0.650	7.64E-08
**OXYS from age P3 to P10**					
ENSRNOG00000056024	Bnip2	BCL2 interacting protein 2	p38	0,69	8,76E-18
ENSRNOG00000004060	Calm1	calmodulin 1	p38	-0,63	1,78E-28
ENSRNOG00000009280	Calm2	calmodulin 2	p38	-0,59	1,33E-31
ENSRNOG00000027690	Camk2b	calcium/calmodulin-dependent protein kinase II beta	p38	-0,48	9,63E-18
ENSRNOG00000013536	Cdc42	cell division cycle 42	p38, JNK	0,25	2,13E-12
ENSRNOG00000011789	Cdon	cell adhesion associated, oncogene regulated	p38	0,42	3,71E-07
ENSRNOG00000019584	Dlk1	delta like non-canonical Notch ligand 1	p38	-1,47	2,88E-05
ENSRNOG00000003977	Dusp1	dual specificity phosphatase 1	p38, JNK	0,32	3,20E-04
ENSRNOG00000004003	Dusp10	dual specificity phosphatase 10	p38, JNK	-1,07	1,80E-07
ENSRNOG00000019822	Gadd45b	growth arrest and DNA-damage-inducible, beta	p38	-1,11	5,81E-06
ENSRNOG00000013090	Gadd45g	growth arrest and DNA-damage-inducible, gamma	p38	-1,43	6,15E-18
ENSRNOG00000008144	Irf1	growth arrest and DNA-damage-inducible, gamma	p38	-1,15	8,32E-07
ENSRNOG00000004437	Map2k6	mitogen-activated protein kinase kinase 6	p38	0,47	4,28E-10

ENSRNOG00000023521	Map3k10	mitogen-activated protein kinase kinase kinase 10	p38, JNK	-0,61	2,06E-47
ENSRNOG00000020773	Map3k11	mitogen-activated protein kinase kinase kinase 11	p38, JNK	-0,24	2,27E-02
ENSRNOG00000014089	Map3k2	mitogen-activated protein kinase kinase kinase 2	ERK1/2, JNK	0,66	5,03E-02
ENSRNOG00000031700	Map3k5	mitogen-activated protein kinase kinase kinase 5	p38	-1,00	7,67E-24
ENSRNOG00000016378	Map3k8	mitogen-activated protein kinase kinase kinase 8	p38	-1,07	2,17E-03
ENSRNOG00000001849	Mapk1	mitogen activated protein kinase 1	ERK1/2	-0,63	1,98E-28
ENSRNOG00000002079	Mapk10	mitogen activated protein kinase 10	JNK	-0,24	1,08E-04
ENSRNOG00000031233	Mapk12	mitogen-activated protein kinase 12	p38	-0,59	3,99E-03
ENSRNOG00000020155	Mapk8	mitogen-activated protein kinase 8	JNK	0,71	5,22E-07
ENSRNOG00000058478	Mapk8ip1	mitogen-activated protein kinase 8 interacting protein 1	JNK	-0,44	1,38E-26
ENSRNOG00000032828	Mapk8ip2	mitogen-activated protein kinase 8 interacting protein 2	p38, JNK	-0,14	7,34E-03
ENSRNOG00000002823	Mapk9	mitogen-activated protein kinase 9	JNK	-0,62	9,89E-25
ENSRNOG00000014832	Mapkapk3	MAPK activated protein kinase 3	p38	-1,34	1,11E-24
ENSRNOG00000001345	Mapkapk5	MAPK activated protein kinase 5	p38	0,31	4,42E-08
ENSRNOG00000010381	Mknk1	MAPK interacting serine/threonine kinase 1	p38, ERK1/2	-0,40	4,02E-03
ENSRNOG00000029028	Mknk2	MAPK interacting serine/threonine kinase 2	p38, ERK1/2	0,27	1,14E-06
ENSRNOG00000023079	Nras	NRAS proto-oncogene, GTPase	ERK1/2	0,52	2,49E-12
ENSRNOG00000001136	Pebp1	phosphatidylethanolamine binding protein 1	ERK1/2	-0,08	6,40E-02
ENSRNOG00000003329	Ppm1d	protein phosphatase, Mg2+/Mn2+ dependent, 1D	p38	0,46	1,69E-09
ENSRNOG00000027839	Ptk2b	protein tyrosine kinase 2 beta	ERK1/2	-0,36	1,25E-04
ENSRNOG00000001068	Rac1	Rac family small GTPase 1	p38, JNK	0,09	2,37E-02
ENSRNOG00000010153	Raf1	Raf-1 proto-oncogene, serine/threonine kinase	ERK1/2	0,59	5,74E-33
ENSRNOG00000014316	Rapgef1	Rap guanine nucleotide exchange factor 1	JNK	0,41	7,13E-12
ENSRNOG00000042411	Rps6ka1	ribosomal protein S6 kinase A1	ERK1/2	0,32	2,64E-03
ENSRNOG00000013194	Rps6ka2	ribosomal protein S6 kinase A2	ERK1/2	-0,38	2,33E-11

ENSRNOG00000006632	Rps6ka3	ribosomal protein S6 kinase A3	ERK1/2	0,21	1,10E-02
ENSRNOG00000004362	Rps6ka5	ribosomal protein S6 kinase A5	p38, ERK1/2	0,53	2,08E-08
ENSRNOG00000002592	Rps6ka6	ribosomal protein S6 kinase A6	ERK1/2	0,54	6,21E-06
ENSRNOG00000020657	Shc1	SHC adaptor protein 1	ERK1/2	-0,46	1,06E-03
ENSRNOG00000018483	Smad1	SMAD family member 1	ERK1/2	0,49	3,41E-15
ENSRNOG00000002749	Spag9	sperm associated antigen 9	p38	0,34	2,90E-06
ENSRNOG00000043451	Spp1	secreted phosphoprotein 1	ERK1/2, JNK	-0,85	2,02E-02
ENSRNOG00000016054	Tab2	TGF-beta activated kinase 1/MAP3K7 binding protein 2	p38	0,20	8,63E-04
ENSRNOG00000001138	Taok3	TAO kinase 3	p38	0,14	1,48E-02
ENSRNOG00000006238	Traf2	Tnf receptor-associated factor 2	p38, JNK	-0,53	2,06E-04
ENSRNOG00000004639	Traf6	TNF receptor associated factor 6	p38	0,54	6,10E-04
ENSRNOG00000012081	Txn1	thioredoxin 1	p38	-0,42	2,05E-17
ENSRNOG00000010838	Araf	A-Raf proto-oncogene, serine/threonine kinase	ERK1/2	-0,24	4,37E-05
ENSRNOG00000010957	Braf	B-Raf proto-oncogene, serine/threonine kinase	ERK1/2	0,32	8,50E-03
ENSRNOG00000023896	Dusp6	dual specificity phosphatase 6	ERK1/2	-1,11	1,25E-65
ENSRNOG00000016611	Hras	HRas proto-oncogene, GTPase	ERK1/2	-0,28	2,12E-09
ENSRNOG00000010176	Map2k1	mitogen activated protein kinase kinase 1	ERK1/2	-1,27	1,79E-60
ENSRNOG00000020005	Map2k2	mitogen activated protein kinase kinase 2	ERK1/2	0,17	2,48E-03
ENSRNOG00000003834	Map2k4	mitogen activated protein kinase kinase 4	JNK	-0,58	5,98E-16
ENSRNOG00000025423	Map3k13	mitogen-activated protein kinase kinase kinase 13	JNK	0,56	5,32E-10

Supplementary Table S3. The list of DEG involved in MAPK signaling pathways (ERK1/2, JNK, and p38) in hippocampus and PFC of OXYS rats at the age of P3 and P10 compared to Wistar rats.

Gene ID	Gene symbol	Gene name	MAPK pathway	log2Fold Change	padj
**In hippocampus at the age of P3**					
ENSRNOG00000016770	Calm2	calmodulin 2	p38	0,12	4,06E-02
ENSRNOG00000029773	Atm	ATM serine/threonine kinase	p38	-0,21	4,77E-02
ENSRNOG00000043451	Spp1	secreted phosphoprotein 1	ERK1/2, JNK	2,29	1,45E-11
ENSRNOG00000016346	Prkcd	protein kinase C delta	ERK1/2	0,72	9,05E-15

ENSRNOG00000020873	Nphs1	NPHS1 adhesion molecule nephrin	JNK	1,77	3,94E-05
ENSRNOG00000010589	Zfp622	zinc finger protein 622	JNK	0,36	8,48E-03
ENSRNOG00000021061	Map4k2	mitogen activated protein kinase kinase kinase kinase 2	JNK	0,18	3,56E-02
ENSRNOG00000028129	Fktn	fukutin	JNK	0,17	4,20E-03
**In hippocampus at the age of P10**					
ENSRNOG00000032828	Mapk8ip2	mitogen-activated protein kinase 8 interacting protein 2	p38, JNK	-0,14	3,33E-04
ENSRNOG00000012081	Txn1	thioredoxin 1	p38	0,16	2,03E-02
ENSRNOG00000043451	Spp1	secreted phosphoprotein 1	ERK1/2, JNK	1,32	2,29E-02
ENSRNOG00000016346	Prkcd	protein kinase C, delta	ERK1/2	0,51	4,76E-06
ENSRNOG00000020005	Map2k2	mitogen activated protein kinase kinase 2	ERK1/2	-0,18	7,99E-03
ENSRNOG00000053583	Mapk3	mitogen activated protein kinase 3	ERK1/2	-0,11	4,18E-02
ENSRNOG00000023896	Dusp6	dual specificity phosphatase 6	ERK1/2	0,23	4,02E-03
ENSRNOG00000028129	Fktn	fukutin	JNK	0,37	2,66E-03
ENSRNOG00000008868	Dusp19	dual specificity phosphatase 19	JNK	0,34	6,28E-03
ENSRNOG00000015134	Map3k12	mitogen activated protein kinase kinase kinase 12	JNK	-0,11	1,23E-02
**In PFC at the age of P3**					
ENSRNOG00000058805	Aida	axin interactor, dorsalization associated	JNK	-0,21	1,41E-02
ENSRNOG00000029773	Atm	ATM serine/threonine kinase	p38	-0,28	3,59E-02
ENSRNOG00000001323	Dusp19	dual specificity phosphatase 19	JNK	0,36	2,26E-02
ENSRNOG00000022554	Fzd10	frizzled class receptor 10	JNK	1,08	3,54E-09
ENSRNOG00000014428	Map3k10	mitogen-activated protein kinase kinase kinase 10	p38	0,33	8,10E-12
ENSRNOG00000010627	Map3k11	mitogen-activated protein kinase kinase kinase 11	p38, JNK	0,34	1,88E-03
ENSRNOG00000009381	Map3k6	mitogen-activated protein kinase kinase kinase 6	p38	0,70	3,83E-02
ENSRNOG00000013612	Map4k2	mitogen-activated protein kinase kinase kinase kinase 2	JNK	0,24	1,18E-02
ENSRNOG00000009562	Mapk8ip1	mitogen-activated protein kinase 8 interacting protein 1	JNK	0,11	3,60E-02
ENSRNOG00000029028	Mknk2	MAPK interacting serine/threonine kinase 2	ERK1/2	0,18	4,68E-03

ENSRNOG00000002516	Mturn	maturin, neural progenitor differentiation regulator homolog	JNK	-0,29	8,35E-03
ENSRNOG00000020713	Myd88	MYD88 innate immune signal transduction adaptor	JNK	0,70	3,63E-03
ENSRNOG00000053200	Nphs1	NPHS1 adhesion molecule, nephrin	JNK	1,58	1,06E-02
ENSRNOG00000001136	Pebp1	phosphatidylethanolamine binding protein 1	ERK1/2	0,10	3,23E-02
ENSRNOG00000016346	Prkcd	protein kinase C delta	ERK1/2	0,80	1,29E-13
ENSRNOG00000027839	Ptk2b	protein tyrosine kinase 2 beta	ERK1/2	0,27	1,39E-02
ENSRNOG00000021117	Rps6ka4	ribosomal protein S6 kinase A4	p38, JNK	0,24	6,45E-03
ENSRNOG00000020657	Shc1	SHC adaptor protein 1	ERK1/2	0,54	2,86E-04
ENSRNOG00000002749	Spag9	sperm associated antigen 9	p38	-0,33	7,00E-03
**In PFC at the age of P10**					
ENSRNOG00000016770	Calm2	calmodulin 2	p38	0,15	2,36E-02
ENSRNOG00000029773	Atm	ATM serine/threonine kinase	p38	-0,28	4,06E-04
ENSRNOG00000000513	Mapk14	mitogen-activated protein kinase 14	p38	-0,20	2,06E-02
ENSRNOG00000013683	S1pr1	sphingosine-1-phosphate receptor 1	ERK1/2	0,21	2,22E-03
ENSRNOG00000023896	Dusp6	dual specificity phosphatase 6	ERK1/2	0,23	4,48E-03
ENSRNOG00000005175	Spp1	secreted phosphoprotein 1	ERK1/2	1,22	1,24E-05
ENSRNOG00000001818	Fgd4	FYVE, RhoGEF and PH domain containing 4	JNK	-0,40	3,17E-03
ENSRNOG00000002516	Mturn	maturin, neural progenitor differentiation regulator homolog	JNK	-0,28	4,46E-05
ENSRNOG00000015750	Wnt7b	Wnt family member 7B	JNK	0,27	2,09E-02
ENSRNOG00000013612	Map4k2	mitogen-activated protein kinase kinase kinase kinase 2	JNK	0,29	7,63E-06
ENSRNOG00000022554	Fzd10	frizzled class receptor 10	JNK	0,34	2,47E-03
